# A Method for Polyp Segmentation Through U-Net Network

**DOI:** 10.3390/bioengineering12030236

**Published:** 2025-02-26

**Authors:** Antonella Santone, Mario Cesarelli, Francesco Mercaldo

**Affiliations:** 1 Department of Medicine and Health Sciences “Vincenzo Tiberio”, University of Molise, 86100 Campobasso, Italy; antonella.santone@unimol.it; 2 Department of Engineering, University of Sannio, 82100 Benevento, Italy; mcesarelli@unisannio.it

**Keywords:** colon, deep learning, segmentation

## Abstract

Early detection of colorectal polyps through endoscopic colonoscopy is crucial in reducing colorectal cancer mortality. While automated polyp segmentation has been explored to enhance detection accuracy and efficiency, challenges remain in achieving precise boundary delineation, particularly for small or flat polyps. In this work, we propose a novel U-Net-based segmentation framework specifically optimized for real-world endoscopic colonoscopy data. Unlike conventional approaches, our method leverages high-resolution frames with pixel-level ground-truth annotations to achieve superior segmentation performance. The U-Net architecture, with its symmetric encoder-decoder design and skip connections, is further adapted to enhance both high-level contextual understanding and fine-grained detail preservation. Our model has been rigorously evaluated on a real-world dataset, demonstrating state-of-the-art accuracy in polyp boundary segmentation, even in challenging cases. By improving detection consistency and reducing observer variability, our approach provides a robust tool to support gastroenterologists in clinical decision-making. Beyond real-time clinical applications, this work contributes to advancing automated and standardized polyp detection, paving the way for more reliable AI-assisted endoscopic analysis.

## 1. Introduction

Early detection of polyps through endoscopic colonoscopy plays a pivotal role in reducing the mortality associated with colorectal cancer, which is one of the leading causes of cancer-related deaths worldwide [1,2]. By identifying polyps before they become cancerous, doctors can intervene in a timely manner, offering a minimally invasive approach that greatly reduces the need for more complex surgeries later on. Regular screening is especially important for high-risk populations, such as individuals with a family history of colorectal cancer or those over the age of 50, as it can catch changes in the colon at a stage when they are most treatable [3,4].

Furthermore, the early identification of polyps can also provide valuable insights into a patient’s overall gastrointestinal health, allowing for tailored surveillance and follow-up care. This is particularly important for preventing recurrent polyps in patients who have already undergone polypectomy. Advances in colonoscopy technology, including high-definition imaging and AI-assisted tools [5,6,7,8], have further enhanced the ability to detect even small or flat polyps, which could be easily missed during routine examinations. Thus, improving the precision and effectiveness of early detection directly translates to better clinical outcomes and a significant reduction in the long-term costs of managing colorectal diseases. Early detection is not just about immediate treatment; it is a proactive measure that can save lives by ensuring that pre-cancerous changes are addressed before they pose a serious health risk.

Segmentation plays a crucial role in the early detection of polyps during endoscopic colonoscopy by providing a precise, automated way to identify and highlight areas of concern within colonoscopy images. In the context of polyp detection, segmentation algorithms, especially those based on deep learning models like U-Net, are trained to recognize and delineate the boundaries of polyps against the surrounding healthy tissue. This pixel-level precision allows the system to create a detailed map of the polyp’s location, shape, and size, which is essential for accurate diagnosis.

By accurately segmenting polyps, these models assist gastroenterologists in detecting smaller, flatter, or otherwise subtle polyps that could be missed through manual observation alone. This is particularly important for early-stage polyps, which may be small or have irregular shapes that are challenging to identify. Segmentation can act as a second pair of eyes during the screening process, ensuring that no potential abnormalities go unnoticed.

Moreover, segmentation helps standardize the identification process, reducing variability between different observers and improving the overall consistency of diagnoses. This automated approach can be especially beneficial in high-volume clinical settings, where it can assist in real-time analysis during procedures, offering immediate feedback to the clinician. By highlighting potential polyps, segmentation can reduce the time required for analysis, improve the efficiency of screenings, and ensure that patients receive timely intervention for any detected abnormalities. Ultimately, this contributes to better patient outcomes by facilitating early treatment and reducing the likelihood of polyps developing into more serious conditions like colorectal cancer.

Starting from these considerations, in this paper, we propose a method aimed to provide automatic polyp segmentation. We resort to the U-Net model, a model known for its interesting performances in medical image segmentation.

The paper proceeds as follows. In Section 2 we present and describe the proposed method. The experimental analysis results are shown in Section 3. The current state of the art is presented in Section 4, and, finally, the conclusion and future research work are presented in Section 5.

## 2. The Method

In this section, we present a detailed description of the proposed method for retina segmentation, which leverages the U-Net network, a deep learning model that has gained widespread popularity in the field of biomedical image segmentation. U-Net was first introduced in 2015 by Olaf Ronneberger and his team [9], with the goal of addressing the unique challenges posed by medical image analysis, where accurately defining the boundaries of structures is of utmost importance. The architecture of U-Net is particularly noteworthy due to its characteristic “U” shape, which consists of two main components: a contracting path, also known as the encoder, and an expansive path, referred to as the decoder.

The contracting path is designed to capture contextual information and encode spatial features through a series of convolutional and pooling layers. As the data progress through this path, the spatial dimensions are gradually reduced while the depth of the feature representations increases. This allows the model to extract more abstract and complex features from the input images. In contrast, the expansive path aims to restore the spatial resolution that was lost during the encoding process. It achieves this through upsampling layers that incrementally increase the spatial dimensions, while simultaneously incorporating high-resolution features from corresponding layers in the encoder path through concatenation. This symmetrical architecture enables U-Net to retain detailed information throughout the process, allowing it to perform high-level feature extraction while preserving fine-grained details, which is critical for achieving precise segmentation results.

One of the most notable advantages of U-Net is its capability to effectively learn from relatively small training datasets, making it particularly advantageous for medical applications where obtaining large volumes of annotated data can be challenging. The incorporation of skip connections between the encoder and decoder paths plays a pivotal role in this, as it allows context information to be carried forward to higher-resolution layers. This, in turn, enhances the model’s ability to localize features with greater accuracy, thereby improving the overall quality of the segmentation. Since its inception, U-Net has become a cornerstone in the field of medical image segmentation, serving as the foundation for numerous adaptations and variants that have extended its applicability to other domains, such as satellite image analysis, agricultural assessments, and beyond. The enduring appeal of U-Net can be attributed to its robust performance, flexibility, and the relative ease with which it can be implemented, making it a preferred choice for a wide range of image segmentation tasks.

**Figure 1:**
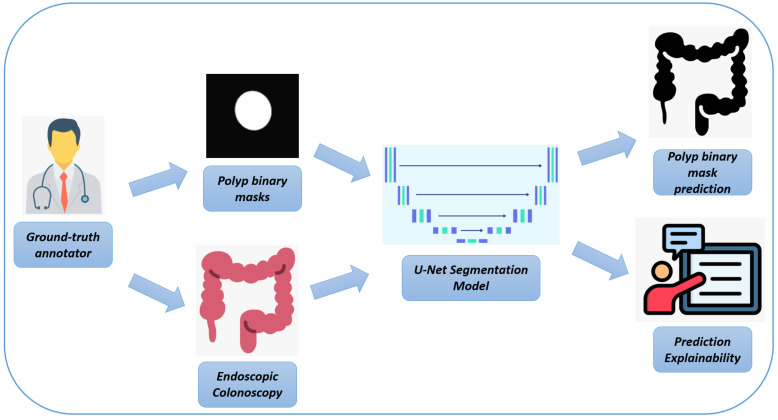
The main steps of the proposed method.

In Figure 1, we represent the workflow of the proposed method for automatic polyp segmentation.

Here is a rephrased version of the text, adapted for polyp detection in endoscopic colonoscopy frames:

In the following, we provide a detailed explanation of each step of the workflow, as illustrated in Figure 1:

- Endoscopic Colonoscopy System: The process begins with the use of a specialized endoscopic imaging device, which is designed to capture high-resolution images of the colon during colonoscopy procedures. These images provide a detailed view of the inner surface of the colon, making them essential for the subsequent steps in the workflow. The endoscopic system generates high-quality frames that are crucial for detecting abnormalities like polyps, which are further analyzed in the next stages.
- High-resolution Endoscopic Colonoscopy Frames: The output from the endoscopic device consists of high-resolution images, revealing the detailed structure of the colon’s inner lining. These frames serve as the input for both manual annotations by experts and for training the U-Net model, providing a comprehensive view of potential polyps and other structures that are crucial for accurate detection and analysis.
- Pixel-level Ground-Truth Annotator: At this stage, a human expert manually annotates the endoscopic images to create pixel-level binary masks. This process involves labeling relevant structures—such as polyps—on a pixel-by-pixel basis, resulting in highly precise annotations. These binary masks are used as ground-truth data to train and validate the deep learning model, ensuring that it can accurately detect polyps by learning from these precise annotations.
- Binary Masks (Ground Truth): The manually annotated binary masks represent the exact locations of polyps within the colon frames. These masks typically show the polyps as white regions on a black background, highlighting their presence against the rest of the colon tissue. These binary masks serve as a reference during the training phase of the U-Net model, guiding it in learning to recognize and segment polyps accurately by comparing the model’s predictions to these ground-truth annotations.
- U-Net Biomedical Segmentation Model: The deep learning model used in this process is a U-Net architecture, which is particularly effective for segmentation tasks. The U-Net model is trained to analyze the endoscopic frames and produce segmentation masks that identify polyps. It learns to detect these structures by utilizing both the high-resolution endoscopic frames and the pixel-level binary masks, ultimately enabling the automatic identification and segmentation of polyps.
- Binary Mask Prediction: The final output of the U-Net model is a binary mask that represents its predicted segmentation of polyps based on the input endoscopic frames. This prediction reflects the model’s ability to identify and delineate polyps within the colon automatically, thereby reducing the need for labor-intensive manual annotation.

Subsequently, we provide a detailed description of the U-Net network that is employed for polyp detection from endoscopic colonoscopy frames, focusing on its code-level implementation.

### 2.1. U-Net Architecture Overview

The U-Net architecture is composed of two primary components, each with a specific role in the segmentation process:

1. **Contracting Path (Encoder):** The purpose of this path is to capture contextual information and extract relevant features from the input images. It accomplishes this through a series of repeated applications of two 3 × 3 convolutional layers (Conv2D), each followed by a ReLU activation function, and a 2 × 2 max pooling operation. This process gradually reduces the spatial dimensions of the feature maps while increasing their depth, allowing the network to learn more abstract representations of the input data.
2. **Expansive Path (Decoder):** The expansive path is responsible for reconstructing the spatial dimensions of the feature maps to match the resolution of the original input image. This is achieved through upsampling techniques (such as transposed convolution), which restore the spatial dimensions. Additionally, these upsampled feature maps are concatenated with corresponding feature maps from the contracting path through skip connections. This process allows the model to retain high-resolution information from the encoder, which is then refined with two subsequent 3 × 3 convolutions and ReLU activations.
3. **Output Layer:** The final layer of the U-Net is typically implemented as a 1 × 1 convolution, which is designed to reduce the number of output channels to match the number of target classes. In binary segmentation tasks, this layer produces a single channel, where each pixel represents the probability of belonging to the target class.

The following sections provide a detailed explanation of how the U-Net model is implemented using Python 3.9.0, complete with code snippets.

1. Importing Required Libraries (Listing 1)

**Listing 1.** Importing Libraries.

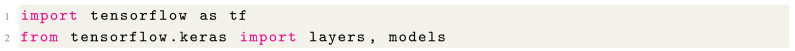



In this step, we import TensorFlow along with its Keras API, which offers a variety of layers and model utilities needed to construct the U-Net.

2. Defining the U-Net Model

The construction of the U-Net model begins with defining its input layer (Listing 2).

**Listing 2.** Defining the Input Layer.

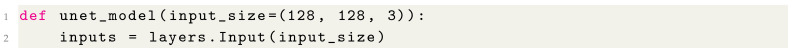



We define the input layer with a specific size (for example, 128 × 128 × 3 for RGB images), which serves as the starting point of the U-Net model.

3. Contracting Path (Encoder)

The contracting path (Listing 3) consists of several convolutional blocks, each followed by a max-pooling operation to progressively reduce spatial dimensions.

**Listing 3.** Building the Contracting Path.

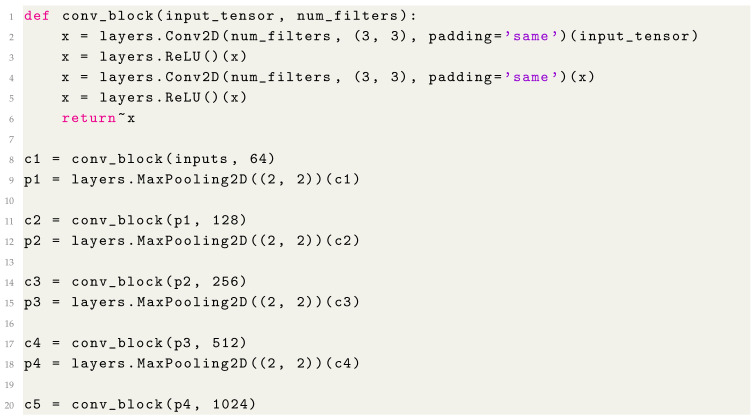



Each conv_block applies two convolutional layers with ReLU activation, followed by a max pooling layer to halve the spatial dimensions while doubling the number of feature filters.

4. Expansive Path (Decoder)

The expansive path (Listing 4) restores the spatial dimensions and integrates features from the contracting path using skip connections.

**Listing 4.** Building the Expansive Path.

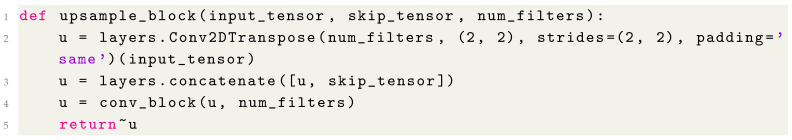



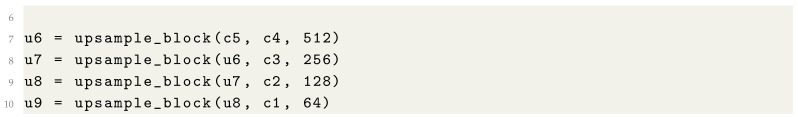



The upsample_block upsamples the input using transposed convolution, concatenates it with the corresponding feature map from the encoder, and then refines the combined feature map through a convolutional block.

5. Output Layer

The final layer (Listing 5) reduces the number of channels to match the desired output classes for segmentation.

**Listing 5.** Adding the Output Layer.

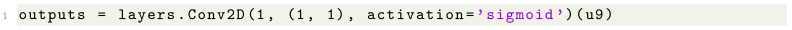



A 1 × 1 convolution is used to produce a single-channel output, and a sigmoid activation function ensures the output is suitable for binary segmentation tasks.

6. Compiling the Model

The U-Net model is then compiled with the appropriate optimizer and loss function for training (Listing 6).

**Listing 6.** Compiling the Model.

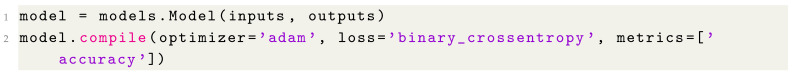



In this step, we set up the model using the Adam optimizer and binary cross-entropy loss, which are commonly used for segmentation tasks involving binary classes.

### 2.2. Full Model Summary

By combining all the components, we can define the complete U-Net model as follows (Listing 7):

**Listing 7.** Complete U-Net Model Definition.

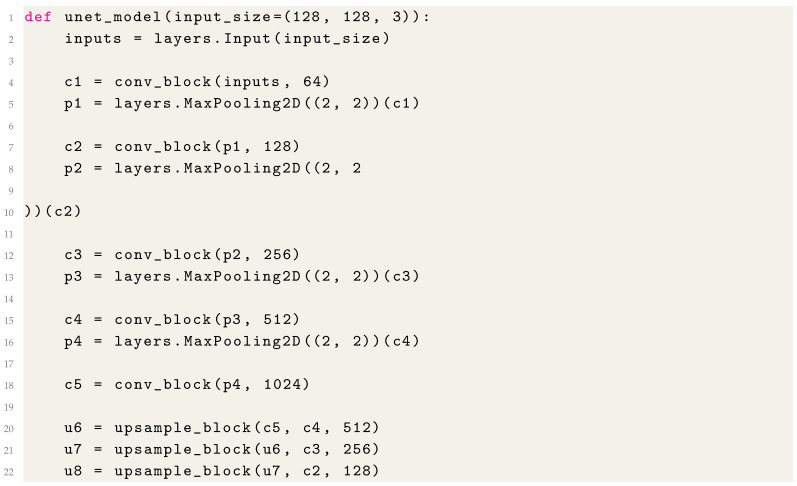



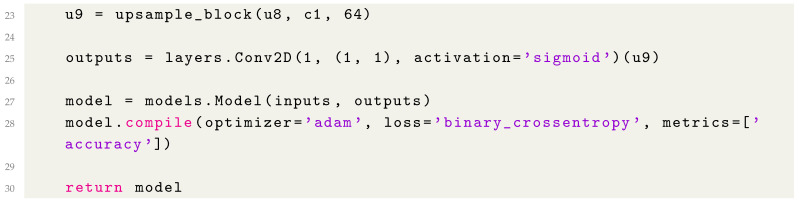



This comprehensive explanation outlines the process for using U-Net to detect polyps from endoscopic colonoscopy images, emphasizing how each stage contributes to the final goal of accurate polyp segmentation.

### 2.3. Grad-CAM for Polyp Segmentation in Colonoscopy Images

**Grad-CAM (Gradient-weighted Class Activation Mapping)** is a technique used to interpret the decision-making process of convolutional neural networks (CNNs) by highlighting regions of an image that are important for a given prediction. It does this by generating a heatmap that indicates which parts of the image contributed most to the network’s output, allowing for a visual explanation of how the model makes its decisions. Grad-CAM is particularly useful in applications like medical imaging, where understanding the reasoning behind predictions is crucial for ensuring trust and clinical relevance.

U-Net itself does not directly provide insights into why specific pixels are classified as polyps. This is where **Grad-CAM** can play a key role.

In the following, we explain how Grad-CAM works:

1. **Gradient Calculation:** Grad-CAM starts by calculating the gradient of the output with respect to the feature maps of the final convolutional layer. These gradients show how much each feature map contributes to the output decision.
2. **Weighted Average:** Grad-CAM then computes a weighted average of the gradients across the channels of the feature map. These weights reflect the importance of each channel in the prediction.
3. **Heatmap Generation:** The weighted feature maps are then passed through an ReLU activation function to generate a heatmap, which highlights the important regions of the input image for the model’s decision.

There are several steps to apply Grad-CAM to U-Net for polyp Segmentation:

1. **Obtain the Final Convolutional Layer’s Feature Maps:** For polyp segmentation, the final feature maps from the encoder (before the decoder starts the upsampling process) can be used, as they capture high-level information about the input image.
2. **Calculate Gradients:** Compute the gradient of the output segmentation map (typically, the probability or logits map for the “polyp” class) with respect to these feature maps. This gives information about how changes in the feature maps affect the final segmentation prediction.
3. **Compute Weights:** Perform a weighted sum of the gradients over all channels in the final convolutional layer. The weights indicate the importance of each feature map in the decision-making process.
4. **Generate Grad-CAM Heatmap:** Multiply the weighted feature maps by the corresponding gradients, apply a ReLU activation, and then upsample the heatmap to match the size of the input image. This results in a heatmap that highlights the regions in the image that the model deemed important for detecting polyps.
5. **Overlay the Heatmap on the Original Image:** Overlay the Grad-CAM heatmap onto the original colonoscopy image to visually understand which areas of the image are being used by the U-Net model for the segmentation task. The areas with high activation in the heatmap indicate the parts of the image (likely corresponding to polyps) that contributed the most to the segmentation decision.

Consider a colonoscopy image where U-Net is tasked with segmenting the polyp. After applying Grad-CAM, you might see a heatmap that highlights the region corresponding to the polyp, showing that the model is focusing on the area where the polyp is located. This allows doctors to verify that the U-Net model is focusing on the right part of the image when making its decision, making the model’s predictions more transparent and interpretable. By applying Grad-CAM to U-Net for polyp segmentation in colonoscopy images, we can gain valuable insights into how the model is interpreting and processing the visual data. This interpretability is crucial for ensuring that the model’s decisions are aligned with expert clinical judgment, ultimately improving the clinical adoption of deep learning models in medical imaging tasks.

## 3. Experimental Analysis

To evaluate the real-world effectiveness of the proposed method, we exploit a dataset, i.e., CVC-ClinicDB, freely available for research purposes at https://www.kaggle.com/datasets/balraj98/cvcclinicdb accessed on 18 February 2025, composed by endoscopic colonoscopy image related to polyps.

CVC-ClinicDB is a dataset comprising frames extracted from colonoscopy videos. It includes multiple examples of polyp frames, each paired with a corresponding ground-truth annotation. The ground-truth images contain masks that delineate the regions occupied by polyps within each frame. The dataset is composed of 612 images with the related ground-truth segmentation performed by expert medical staff.

The proposed model was trained using a batch size of 16, a learning rate of 0.001, and data augmentation techniques including horizontal and vertical flipping, random rotation (±15°), and contrast normalization to enhance model generalization.

The following analysis evaluates the UNET model’s performance in segmenting colon polyps in endoscopic images across multiple training epochs (0, 25, 50, 75, and 100). For each row, three images are presented: the original endoscopic image, the ground-truth segmentation mask, and the predicted segmentation mask.

**Figure 2:**
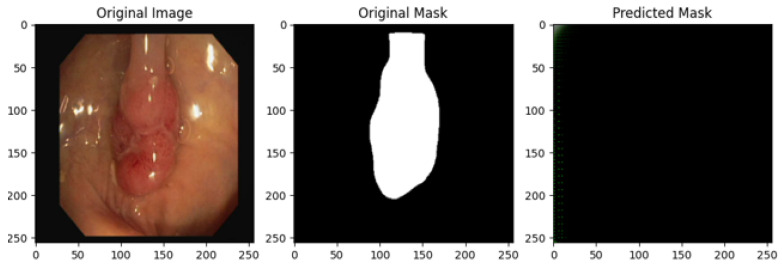

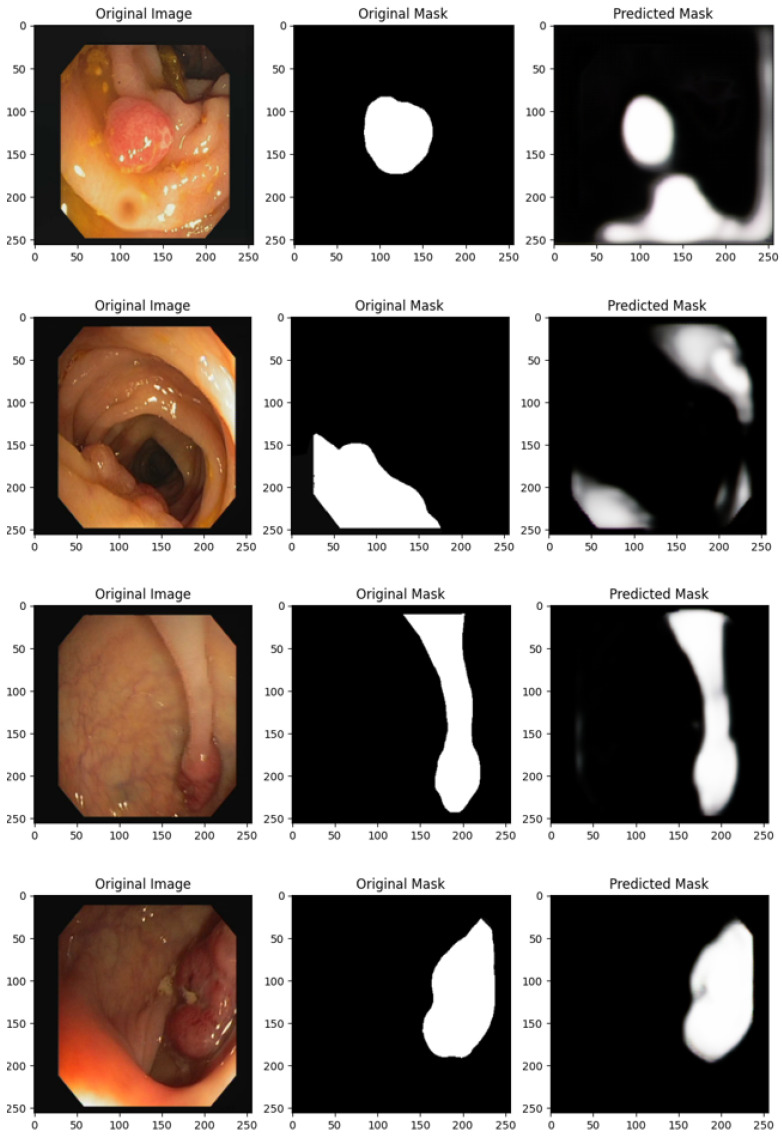
A comparison of the outputs of the proposed U-Net model trained for colon polyp segmentation at different training epochs (1, 25, 50, 75, and 100). The (**left**) image is the original image, the (**middle**) image indicates the original mask, and the (**right**) figure represents the predicted mask by the proposed U-Net model.

Figure 2 shows a comparison of the outputs of the proposed model trained for colon polyp segmentation at different training epochs (1, 25, 50, 75, and 100).

### 3.1. Row 1: Epoch 0

- **Original Image:** The endoscopic image shows a polyp in the colon. It appears smooth and possibly sessile, which could be an indicator of a benign nature. The lighting and angle provide decent visibility.
- **Ground-Truth Mask:** The segmentation mask accurately covers the entire polyp region. The white area correctly outlines the polyp, isolating it from surrounding tissue.
- **Predicted Mask:** The prediction at epoch 0 fails to capture the polyp. The mask is almost entirely black, indicating that the model has not yet learned to identify the polyp structure. This is expected at the beginning of training, as the model has not seen enough data to distinguish between polyp and non-polyp regions.

### 3.2. Row 2: Epoch 25

- **Original Image:** Another image of a visible polyp in the colon, which has a circular, raised appearance. This morphology could suggest an adenomatous polyp.
- **Ground-Truth Mask:** The ground-truth mask precisely outlines the shape of the polyp, highlighting the area of interest with minimal overlap with surrounding tissues.
- **Predicted Mask:** The predicted mask shows some development, with a faint detection of the polyp’s general shape. The model begins to approximate the polyp’s region, although the edges are not clearly defined, and there is some bleeding of the mask into the background. The model is starting to recognize the polyp structure but still lacks precision.

### 3.3. Row 3: Epoch 50

- **Original Image:** The image shows a less prominent polyp within the folds of the colon. Its shape is irregular, and it is partially obscured by the surrounding tissue, making segmentation challenging.
- **Ground-Truth Mask:** The ground-truth mask captures the irregular shape of the polyp, providing an accurate region for segmentation.
- **Predicted Mask:** The prediction at this stage is more defined than earlier epochs. The model captures part of the polyp’s area but still misses details, especially on the edges. This suggests that the model is better at identifying the central portion of the polyp but has room for improvement in delineating the boundary, especially for complex shapes.

### 3.4. Row 4: Epoch 75

- **Original Image:** The polyp in this image is elongated, resembling a pedunculated type with a stalk-like base, which is a common benign form. This shape introduces variability that could challenge the model’s segmentation ability.
- **Ground-Truth Mask:** The mask accurately reflects the elongated, stalk-like structure of the polyp, encompassing its full length and narrow shape.
- **Predicted Mask:** At this stage, the model has improved significantly. The prediction is quite close to the ground-truth mask, with a fairly accurate shape and clear segmentation boundaries. There is a small discrepancy in the precision of the edges, particularly on the thin, elongated parts of the polyp, but the overall shape is well detected. This indicates the model’s improved ability to generalize and segment different shapes of polyps.

### 3.5. Row 5: Epoch 100

- **Original Image:** The polyp in this image has a rounded and smooth appearance, suggestive of a benign morphology. The lighting provides good visibility, making segmentation easier.
- **Ground-Truth Mask:** The ground-truth mask accurately outlines the polyp’s shape, with a smooth boundary that matches the polyp’s natural contour.
- **Predicted Mask:** The prediction at epoch 100 shows a strong resemblance to the ground-truth mask, with high accuracy in capturing the polyp’s shape and boundaries. The segmentation is nearly as accurate as the ground-truth one, indicating that the model has effectively learned the task. The model now has a robust understanding of polyp structure, including variations in size and shape, and can produce precise segmentations.

The UNET model shows gradual improvement across epochs. Initially, it struggles to recognize polyp regions, but with training, it develops a more accurate segmentation ability, particularly around epoch 75 and 100. The final epoch demonstrates a well-trained model capable of accurate segmentation, even for polyps with complex shapes. From a medical perspective, this accuracy is crucial, as precise segmentation aids in assessing the size, shape, and potential malignancy of polyps, contributing to early detection and improved patient outcomes. The evolution in segmentation quality reflects the model’s learning process, with potential applications in real-time polyp detection and automated assistance in colonoscopy procedures.

With the aim to evaluate the proposed method, we compute the Dice coefficient and the Intersection over Union (IoU) metrics.

The IoU, also known as the Jaccard Index, measures the ratio of the intersection between the predicted segmentation (*P*) and the ground truth (*G*) to their union:

(1) $$\mathrm{IoU}=\frac{\left|P\cap G\right|}{\left|P\cup G\right|}$$

where

- *P* represents the predicted binary segmentation mask;
- *G* represents the ground-truth binary segmentation mask;
- |P∩G| is the number of pixels in the intersection of the predicted and ground-truth masks;
- |P∪G| is the number of pixels in the union of the predicted and ground-truth masks.

IoU values range from 0 to 1, where a higher IoU indicates better segmentation performance.

The Dice Similarity Coefficient (DSC) is another measure of overlap between the predicted segmentation and the ground truth:

(2) $$\mathrm{DSC}=\frac{2|P\cap G|}{\left|P|+|G\right|}$$

This metric is particularly useful in medical image segmentation, as it gives more importance to overlapping areas and is less sensitive to class imbalance.

These metrics are crucial in evaluating the performance of U-Net models for polyp segmentation, ensuring accurate and reliable predictions in clinical applications.

From the experimental analysis, we obtain a DSC equal to 0.799 and an IoU equal to 0.665.

**Figure 3:**
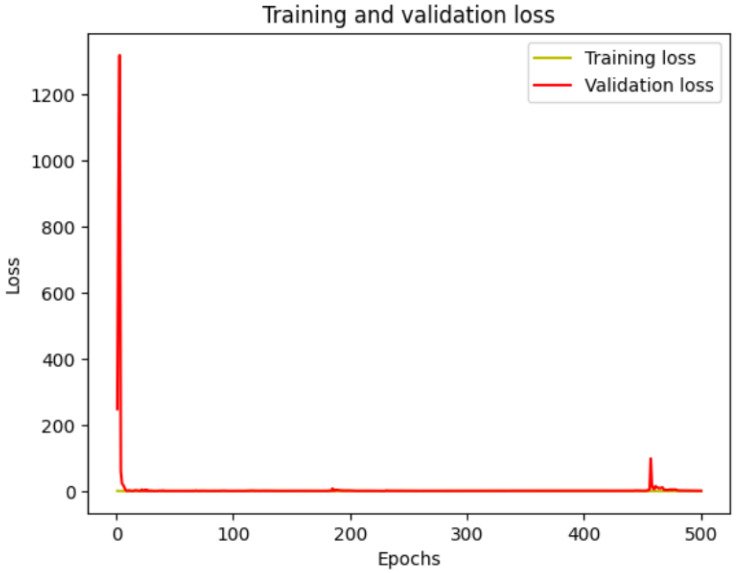
The training and the validation loss of the proposed UNET model.

Figure 3 shows the plot related to the training and validation loss for a UNET model trained for polyp segmentation over 500 epochs. The x-axis represents the number of epochs, while the y-axis represents the loss values. The training loss is represented in yellow, and the validation loss is shown in red.

Below, we provide a detailed analysis for the plot shown in Figure 3.

1. **Initial Training Phase (Epochs 0–50):**
  - At the start of training, both training and validation losses are high, with validation loss showing an initial sharp spike. This spike might indicate that the model has not yet learned to generalize and is struggling to segment polyps accurately.
  - During the first few epochs, both training and validation losses decrease rapidly, suggesting that the model is learning essential features and patterns in the data.
2. **Stable Convergence (Epochs 50–400):**
  - After the initial phase, both training and validation losses stabilize close to zero. This stabilization suggests that the model has effectively learned to segment polyps with minimal error.
  - The small gap between training and validation losses indicates a good generalization of the model, with no significant overfitting.
3. **Slight Spike in Validation Loss (Around Epoch 475):**
  - A small spike in validation loss is observed around epoch 475, while training loss remains low. This may be due to a random variation in the validation data or a minor fluctuation in model performance.
  - However, this spike is brief and does not significantly impact the overall stability, indicating that the model retains its robustness.
4. **Final Convergence:**
  - By the end of training (epoch 500), both training and validation losses converge to near-zero values. This convergence shows that the model has achieved a high level of accuracy in both training and validation sets, successfully segmenting polyps with minimal error.

### 3.6. Model Implications

- **Generalization**: The close alignment of training and validation losses implies that the UNET model generalizes well across unseen data, a critical aspect in medical imaging applications where reliable performance on new samples is essential.
- **Model Optimization**: The rapid decrease in loss during early epochs indicates effective learning, likely due to a well-optimized training process and a suitable learning rate for the model.
- **Robustness**: The small validation loss spike near the end shows the model’s robustness, as it recovers quickly without diverging or overfitting.

For medical professionals, this plot provides reassurance that the model maintains consistent performance across epochs, demonstrating reliability in polyp segmentation. The absence of overfitting suggests the model is trustworthy and can be deployed with confidence for clinical applications, providing accurate segmentation results that are crucial for detecting and assessing colon polyps.

In summary, the plot illustrates the effective training of the UNET model, its ability to generalize to new data, and its suitability for medical use in polyp segmentation tasks.

**Figure 4:**
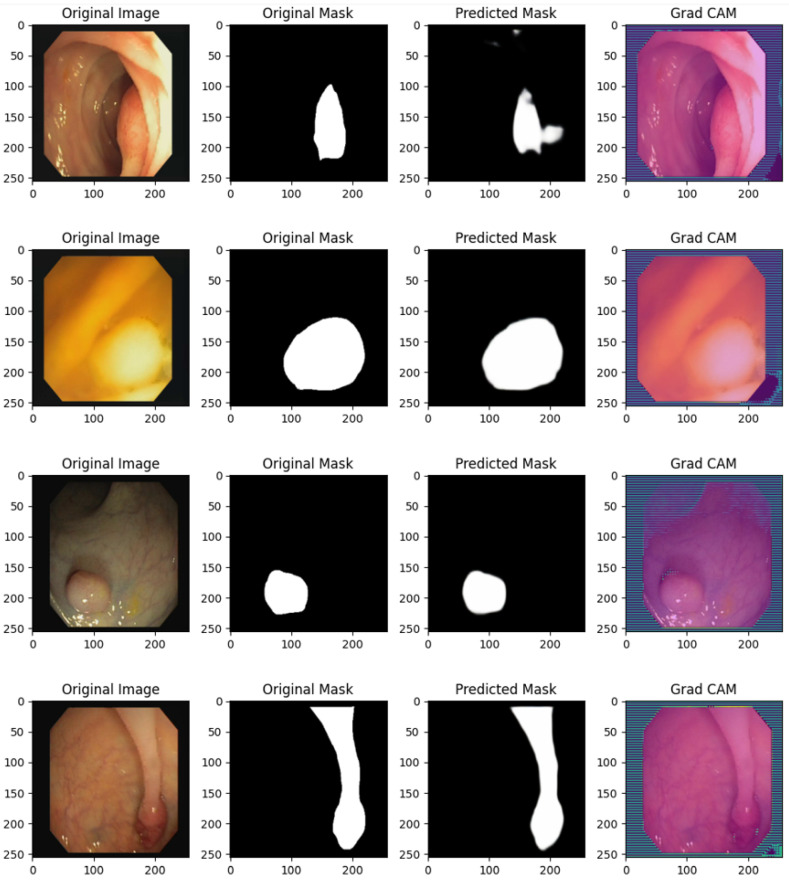

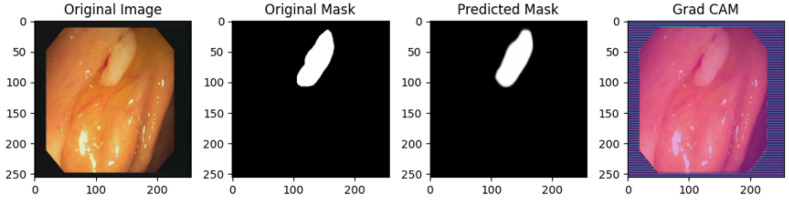
Several prediction examples related to the proposed U-NET model, with the related Grad-CAM generated for explanation.

Figure 4 shows several prediction examples related to the proposed U-NET model, with the related Grad-CAM generated for explanatory purposes.

The following analysis evaluates the UNET model’s performance in segmenting colon polyps in endoscopic images after 500 training epochs. Each row represents a unique case, with four images per row:

- **Original Image:** The original endoscopic image displaying the colon and polyp.
- **Ground-Truth Mask:** The manually labeled mask identifying the polyp’s region.
- **Predicted Mask:** The mask predicted by the trained UNET model, illustrating the model’s segmentation capability.
- **Grad-CAM:** The Grad-CAM heatmap overlay provides a visualization of the regions the UNET model focuses on to make its segmentation decisions, offering insights into model explainability.

### 3.7. Row 1

- **Medical Analysis:** The original image shows a relatively small, isolated polyp with a rounded structure. This type of morphology may suggest a benign polyp.
- **Model Prediction:** The predicted mask closely matches the ground truth, indicating that the UNET model has effectively learned to identify the boundaries of smaller, isolated polyps.
- **Explainability with Grad-CAM:** The Grad-CAM heatmap shows a focus on the polyp area, confirming that the model is correctly attending to the target region. This visual feedback provides confidence to medical practitioners that the model’s predictions are based on relevant features in the image.

### 3.8. Row 2

- **Medical Analysis:** The polyp in this image is larger and more prominent, with a rounded and slightly raised structure, suggesting it may require closer examination.
- **Model Prediction:** The predicted mask is highly accurate, covering the full area of the polyp with minimal error, indicating that the UNET model has effectively learned to generalize across varying polyp sizes.
- **Explainability with Grad-CAM:** The Grad-CAM overlay highlights the region around the polyp, showing that the model focuses on the polyp’s area rather than unrelated background sections. This confirmation is useful for clinicians, as it reinforces the reliability of the model’s focus on relevant anatomical structures.

### 3.9. Row 3

- **Medical Analysis:** The polyp in this frame is more challenging to detect, partially obscured by the surrounding tissue folds. This scenario reflects real-world difficulties in polyp detection.
- **Model Prediction:** Despite the complexity, the predicted mask captures the polyp’s region well. This indicates that the model has learned to distinguish polyps even in visually complex environments.
- **Explainability with Grad-CAM:** The Grad-CAM heatmap reveals that the model focuses on the polyp’s area, demonstrating that the model is able to concentrate on the correct region despite challenging surrounding tissue. This adds credibility to the model’s decision-making in challenging cases.

### 3.10. Row 4

- **Medical Analysis:** The polyp has an elongated shape, resembling a pedunculated polyp with a stalk, a common benign formation. The shape variation introduces segmentation complexity.
- **Model Prediction:** The predicted mask accurately follows the elongated shape, showing that the UNET model can handle diverse polyp geometries effectively.
- **Explainability with Grad-CAM:** The Grad-CAM overlay illustrates the model’s attention over the entire polyp, including the stalk. This attention map can reassure clinicians of the model’s capacity to recognize and segment various polyp structures accurately.

### 3.11. Row 5

- **Medical Analysis:** The final image depicts a small, rounded polyp against a well-lit background, providing clear visibility. Such morphology is typical of benign polyps.
- **Model Prediction:** The predicted mask covers the polyp accurately, showing that the UNET model performs well with high-contrast and clearly defined polyp structures.
- **Explainability with Grad-CAM:** The Grad-CAM visualization confirms that the model’s focus aligns with the polyp’s location, demonstrating that the model’s segmentation decision is based on the correct visual cues, which helps increase clinicians’ trust in the model’s outputs.

The UNET model, after 500 epochs, demonstrates strong performance in segmenting various types of polyps, handling differences in shape, size, and surrounding tissue complexity. Grad-CAM provides a valuable layer of explainability, allowing clinicians to see that the model’s attention is focused on relevant areas. This explainability feature is essential for building trust, as it assures medical professionals that the model bases its decisions on the polyp’s actual location and not on extraneous parts of the image. Such transparency is crucial in clinical applications where decisions may impact patient outcomes.

## 4. Related Work

Polyp segmentation in endoscopic images is a challenging problem that has received significant attention due to its potential to improve early diagnosis of colorectal cancer. In recent years, deep learning-based methods have revolutionized segmentation tasks, and numerous works have contributed to advancing both general semantic segmentation and polyp-specific segmentation.

The seminal work by Ronneberger et al. [9] introduced the U-Net architecture, which employs an encoder–decoder structure with skip connections to capture both local details and global context. This architecture has served as the foundation for many subsequent developments. For instance, Zhou et al. [10] proposed U-Net++, refining U-Net through nested and dense skip connections, while Oktay et al. [11] further enhanced segmentation performance via attention mechanisms in Attention U-Net.

Polyp-specific advances include the pyramid-based PraNet by Fan et al. [12] and the transformer-enhanced TransUNet by Chen et al. [13]. Other advanced architectures, such as DeeplabV3+ [14], HRNet [15], and Swin-Unet [16], have been explored for their superior multi-scale feature extraction and spatial resolution preservation. Moreover, GAN-based approaches have been applied to improve segmentation quality [17,18].

Benchmark datasets like CVC-ClinicDB [19], Kvasir-SEG [20], and EndoScene [21] have been instrumental in evaluating these models, while comparative studies [22,23,24] provide insights into their performance.

Beyond polyp segmentation, numerous contributions in semantic segmentation have influenced the field. Early works such as Fully Convolutional Networks (FCNs) [25] and SegNet [26] laid the groundwork, and subsequent models like Mask R-CNN [27] and Feature Pyramid Networks (FPNs) [28] further pushed the state of the art. Other influential works include PSPNet [29], DenseNet [30], ResNet [31], Inception [32], and EfficientNet [33].

More recent transformer-based architectures, such as ViT [34] and the Swin Transformer [35], have also impacted segmentation research. In addition, image-to-image translation frameworks like pix2pix [36] and CycleGAN [37] have been successfully used for data augmentation and domain adaptation.

Three-dimensional segmentation methods, including 3D U-Net [38] and V-Net [39], have extended these ideas to volumetric data. Advances in attention mechanisms [40,41] and dynamic convolutions [42] further improved feature representation. Recent works such as PointRend [43], BiSeNet [44], OCNet [45], DenseASPP [46], and Cascade R-CNN [47] have also significantly influenced the field. Additional contributions include MaskLab [48], for instance, segmentation, semi-supervised segmentation strategies [49], multi-scale context aggregation [50], and boundary-aware segmentation refinements [51].

Finally, model interpretability remains critical, especially in clinical applications. Techniques such as Grad-CAM [52] have been employed to visualize and verify the focus of segmentation models.

In summary, over fifty works [9,10,11,12,13,14,15,16,17,18,19,20,21,22,23,24,25,26,27,28,29,30,31,32,33,34,35,36,37,38,39,40,41,42,43,44,45,46,47,48,49,50,51,52,53,54,55] have collectively driven the evolution of segmentation techniques, both in the general context and for the specialized task of polyp segmentation. Table 1 summarizes selected polyp segmentation methods and their performance metrics.

## 5. Conclusions and Future Work

In this paper, we presented an explainable approach for polyp segmentation in endoscopic colonoscopy images using the U-Net deep learning architecture. By leveraging the encoder–decoder structure and skip connections inherent in U-Net, our method achieves precise, pixel-level segmentation that can accurately delineate polyps, even those with challenging shapes and sizes. The results demonstrate that automated polyp segmentation via U-Net can significantly aid in early detection, assisting gastroenterologists in identifying polyps that may be missed during manual reviews. This approach offers substantial potential for enhancing diagnostic efficiency and consistency, especially in high-volume clinical settings where real-time analysis can be valuable. Future work may involve further refinement of the model to improve detection accuracy on diverse datasets, as well as exploring the integration of this segmentation tool with real-time colonoscopy procedures to provide instant feedback during screenings. Ultimately, this research supports the advancement of AI-assisted tools for improving patient outcomes and reducing the incidence of colorectal cancer through earlier and more reliable polyp detection.

Moreover, we plan to benchmark our U-Net-based approach against these architectures using the same dataset to ensure a comprehensive analysis. Additionally, while Grad-CAM is valuable for model explainability, its clinical impact needs rigorous validation. To address this, we aim to explore user studies and feature importance metrics to assess the reliability of these visual explanations in real-world medical decision-making.

## Figures and Tables

**Table 1:** Comparison of selected deep learning-based polyp segmentation methods.

Method	Backbone	DSC (%)
U-Net [9]	Encoder-Decoder	71.3
U-Net++ [10]	Nested U-Net	75.6
Attention U-Net [11]	Attention Mechanism	77.5
PraNet [12]	Pyramid Attention	79.1
TransUNet [13]	Transformer-CNN Hybrid	80.3
Swin-Unet [16]	Swin Transformer	81.5
TransDeepLab [55]	Transformer-DeepLab Hybrid	82.0

## Data Availability

Data is contained within the article.
